# Activation Mechanism of Fe^2+^ in Pyrrhotite Flotation: Microflotation and DFT Calculations

**DOI:** 10.3390/molecules29071490

**Published:** 2024-03-27

**Authors:** Qiang Song, Xiong Tong, Pulin Dai, Xian Xie, Ruiqi Xie, Peiqiang Fan, Yuanlin Ma, Hang Chen

**Affiliations:** Faculty of Land Resource Engineering, Kunming University of Science and Technology, Kunming 650093, China; song8023qiang@163.com (Q.S.); kgxiongtong@163.com (X.T.); dpl973624@163.com (P.D.); kgxianxie@126.com (X.X.); ruiqixie@kust.edu.cn (R.X.); fanpeiqiang518@163.com (P.F.); yuanlinma0423@163.com (Y.M.)

**Keywords:** pyrrhotite flotation, activator, DFT

## Abstract

In industrial manufacturing, pyrrhotite(Fe_1−x_S), once depressed, is commonly activated for flotation. However, the replacement of CuSO_4_ is necessary due to the need for exact control over the dosage during the activation of pyrrhotite, which can pose challenges in industrial settings. This research introduces the use of FeSO_4_ for the first time to efficiently activate pyrrhotite. The impact of two different activators on pyrrhotite was examined through microflotation experiments and density functional theory (DFT) calculations. Microflotation experiments confirmed that as the CuSO_4_ dosage increased from 0 to 8 × 10^−4^ mol/L, the recovery of pyrrhotite initially increased slightly from 71.27% to 87.65% but then sharply decreased to 16.47%. Conversely, when the FeSO_4_ dosage was increased from 0 to 8 × 10^−4^ mol/L, pyrrhotite’s recovery rose from 71.27% to 82.37%. These results indicate a higher sensitivity of CuSO_4_ to dosage variations, suggesting that minor alterations in dosage can significantly impact its efficacy under certain experimental conditions. In contrast, FeSO_4_ might demonstrate reduced sensitivity to changes in dosage, leading to more consistent performance. Fe ions can chemically adsorb onto the surface of pyrrhotite (001), creating a stable chemical bond, thereby markedly activating pyrrhotite. The addition of butyl xanthate (BX), coupled with the action of Fe^2+^ on activated pyrrhotite, results in the formation of four Fe-S bonds on Fe^2+^. The proximity of their atomic distances contributes to the development of a stable double-chelate structure. The S 3p orbital on BX hybridizes with the Fe 3d orbital on pyrrhotite, but the hybrid effect of Fe^2+^ activation is stronger than that of nonactivation. In addition, the Fe-S bond formed by the addition of activated Fe^2+^ has a higher Mulliken population, more charge overlap, and stronger covalent bonds. Therefore, Fe^2+^ is an excellent, efficient, and stable pyrrhotite activator.

## 1. Introduction

Pyrrhotite is one of the most abundant iron sulfide ores and is usually associated with sulfide minerals, such as chalcopyrite, pyrite, and pentlandite [1,2,3]. Currently, flotation is the most common commercial process for obtaining pyrrhotite with high efficiency. This mineral is often depressed before entering the tailings during flotation, due to its slow flotation rate, which aids in separating other sulfide minerals [4,5]. Therefore, pyrrhotite needs to be activated for purification after the depressant process [6,7,8].

The activation of depressed pyrrhotite has been the subject of extensive research. Meng et al. revealed that sulfuric acid can enhance the floatability and hydrophobicity of pyrrhotite, effectively activating it for collection [9]. However, due to its high corrosiveness and resource-intensive nature, sulfuric acid can lead to equipment degradation and environmental issues [10]. Consequently, research has shifted towards metal ion activators, which require lesser quantities and cause minimal equipment corrosion. Currently, Cu^2+^ is widely used as a metal activator for separating these depressed iron sulfide ores [11,12]. Various studies have shown that Cu^2+^ activates arsenopyrite under alkaline conditions through the exchange and adsorption of Cu^2+^ ions on the mineral surface [13,14]. Additionally, the interaction between copper hydroxide and xanthate forms basic copper xanthate, promoting preferential adsorption and enhancing xanthate adsorption [15]. Recent studies have also revealed that Cu^2+^ can establish π-backbonding with the collector after adsorption on these iron sulfide minerals, augmenting the interaction between Cu^2+^ and the collector and aiding mineral activation [16]. Nevertheless, activating pyrrhotite with Cu^2+^ necessitates precise dosage control, posing challenges in industrial applications. Therefore, it is necessary to study the use of new activators as alternatives to Cu^2+^, such as Fe^2+^ ions, which are widely used in chemical activations in other fields [17,18,19,20,21,22]. In the study by Cao et al., it was found that the utilization of Fe^2+^ can enhance the adsorption of salicylhydroxamic acid on cassiterite surfaces, thereby achieving improved cassiterite recovery [23]. However, such effects have not been reported for the activation of pyrrhotite flotation, and the activation effect of Fe^2+^ on pyrrhotite is still a “black box”.

DFT simulations have proven to be a highly effective tool in modeling the surface microstructures and adsorption mechanisms of minerals [24,25,26,27]. Through DFT, researchers can gain valuable insights into the atomic- or electronic-level interactions between mineral surfaces and different reagents. This enables accurate prediction and a thorough understanding of the complex dynamics at play.

In this study, we discovered that Fe^2+^ is an excellent activator of pyrrhotite based on the formation of π-backbonding interactions between the collector and metal ions [14]. DFT simulation was used to investigate the interaction of Fe^2+^ on the pyrrhotite (001) surface, and the adsorption characteristics of BX on this surface before and after activation were studied. The stability of Fe^2+^ in pyrrhotite flotation was revealed by combining microflotation tests and adsorption experiments, providing a theoretical basis for the efficient recovery of pyrrhotite.

## 2. Results and Discussion

### 2.1. XRD Analysis

**Figure 1:**
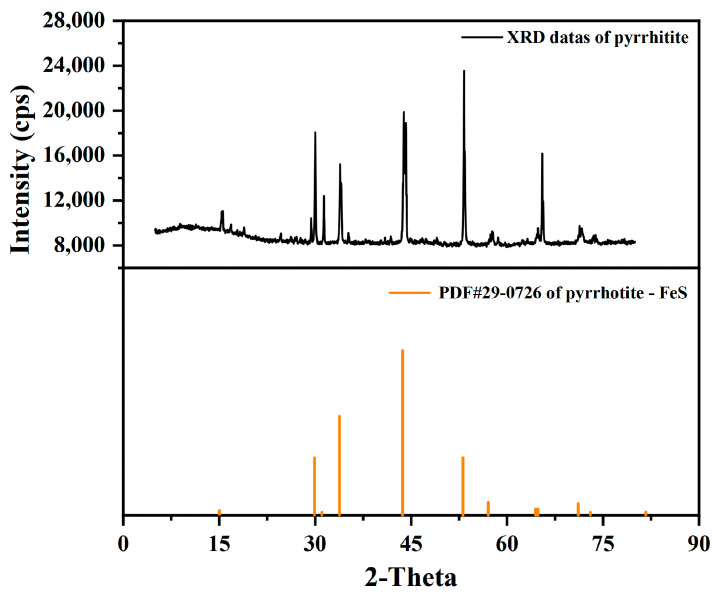
XRD patterns of pure pyrrhotite samples.

The pure monoclinic pyrrhotite sample was analyzed by chemical element analysis and X-ray diffraction, revealing that its Fe and S contents were 59.54 wt % and 40.16 wt %, respectively, and its Cu content was 0.42 wt %. As shown in Figure 1, the purity of pyrrhotite was more than 98%, with only a trace of impurities. It is clear that the sample had no discernible impurity peaks with high purity, meeting the test requirements.

### 2.2. Microflotation for Pyrrhotite

**Figure 2:**
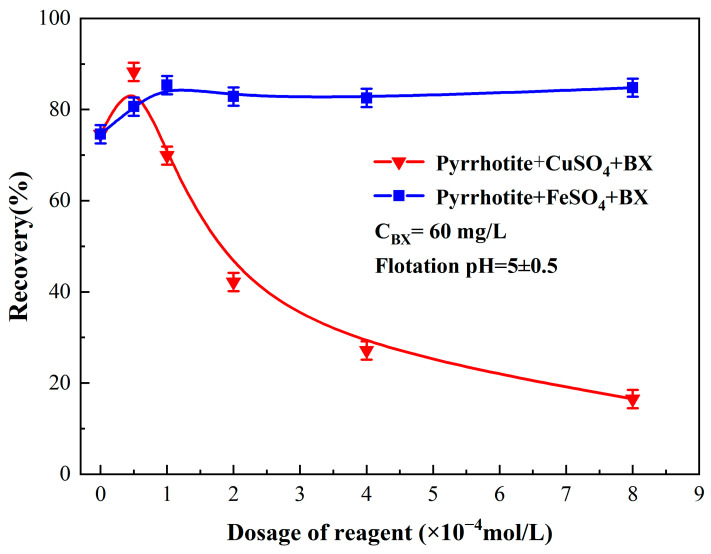
Effect of activators FeSO_4_ and CuSO_4_ on flotation performance for pyrrhotite.

Meng et al. studied the effect of sulfuric acid on the flotation performance of oxidized pyrrhotite and found that BX = 60 mg/L and a pH adjusted to 5.0 had the best collection effect on pyrrhotite [9]. Therefore, this experiment chose BX = 60 mg/L and a pH of 5.0. Limiting other conditions, the effects of Fe^2+^ and Cu^2+^ on pyrrhotite were studied separately. The flotation performance of a single mineral with CuSO_4_ and FeSO_4_ as activators and a slurry pH of 5 is presented in Figure 2.

As depicted in Figure 2, the recovery of pyrrhotite initially rose marginally from 71.27% to 87.65% with an increase in CuSO_4_ dosage from 0 to 8 × 10^−4^ mol/L but subsequently experienced a steep decline to 16.47% as the dosage continued to increase. In contrast, the recovery of pyrrhotite increased consistently to 82.37% from 71.27% when the FeSO_4_ dosage was augmented from 0 to 8 × 10^−4^ mol/L. These findings demonstrate a greater sensitivity of CuSO_4_ to dosage variations, indicating that minor changes in CuSO_4_ dosage can considerably influence its effectiveness under specified experimental conditions. Conversely, FeSO_4_ appears to exhibit less sensitivity to dosage alterations, leading to more stable performance. Thus, FeSO_4_ presents itself as a promising alternative activator to CuSO_4_ for industrial use. In the follow-up study, we mainly studied the effect of Fe^2+^ on pyrrhotite.

### 2.3. Activation of Fe^2+^ on the Pyrrhotite (001) Surface

**Figure 3:**
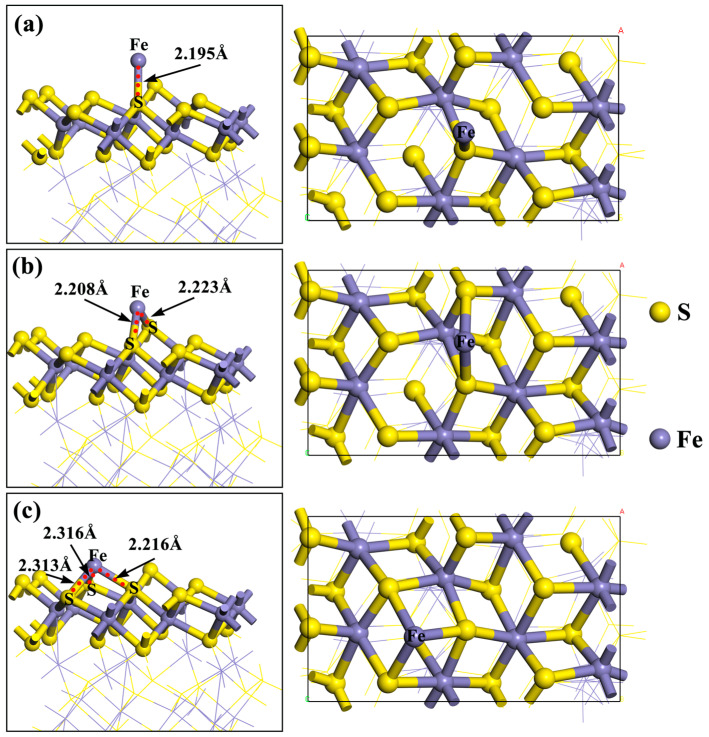
Adsorption model of Fe^2+^ on pyrrhotite (001): (**a**) top position, (**b**) bridge position, and (**c**) meta position.

To directly compare and verify the activation effect of Fe^2+^ on the surface of pyrrhotite, a model representing Fe^2+^ on the pyrrhotite (001) surface was developed. In this modeling process, the top surface S of pyrrhotite (001) was selected as the adsorption site. The geometrically optimized model of this adsorption site was determined through optimization tests, as shown in Figure 3 below.

As shown in Figure 3, in the top position adsorption structure, the adsorption energy between Fe^2+^ and the pyrrhotite surface S is −193.1 kJ/mol, and the atomic distance between Fe^2+^ and the surface S of pyrrhotite is 2.195 Å, which is less than the maximum atomic radius of 2.6Å between Fe^2+^ and S. In the bridge position adsorption structure, the adsorption energy between Fe^2+^ and the pyrrhotite surface S is −179.6 kJ/mol, and the atomic distances between Fe^2+^ and the surface S of pyrrhotite are 2.208 Å and 2.223Å. In the meta position adsorption structure, the adsorption energy between Fe^2+^ and the pyrrhotite surface S is only −171.3 kJ/mol. Compared with the three adsorption structures, the top adsorption site has the largest adsorption energy for Fe^2+^, and the atomic distance between Fe^2+^ and the S atom on the surface of pyrrhotite is the shortest. Therefore, the top adsorption position was selected as the adsorption site for subsequent experimental research. In addition, the three adsorption structures all indicate that Fe^2+^ chemically interacts with the S atom on the surface of pyrrhotite and that Fe^2+^ can chemically adsorb on the surface of pyrrhotite and form a chemical bond to form a relatively stable structure on the surface.

### 2.4. DOS analysis for Fe ions of Pyrrhotite (001)

The definition of the atomic magnetic moment is given by Equation (1), where its magnitude is equal to the difference between the integrated DOS for spin-up and spin-down of Fe d orbitals below the Fermi level.

(1) $$m=\left[\int_{E_{0}}^{E_{f}}DOS\left(spin\;up\right)dE-\int_{E_{0}}^{E_{f}}DOS\left(spin\;down\right)dE\right]\mu_{B}$$

where m is the magnetic moment; DOS (spin-up) and DOS (spin-down) denote the DOS for spin-up and spin-down states, respectively. The integration limits correspond to the DOS at the Fermi level and in the low-price band.

**Figure 4:**
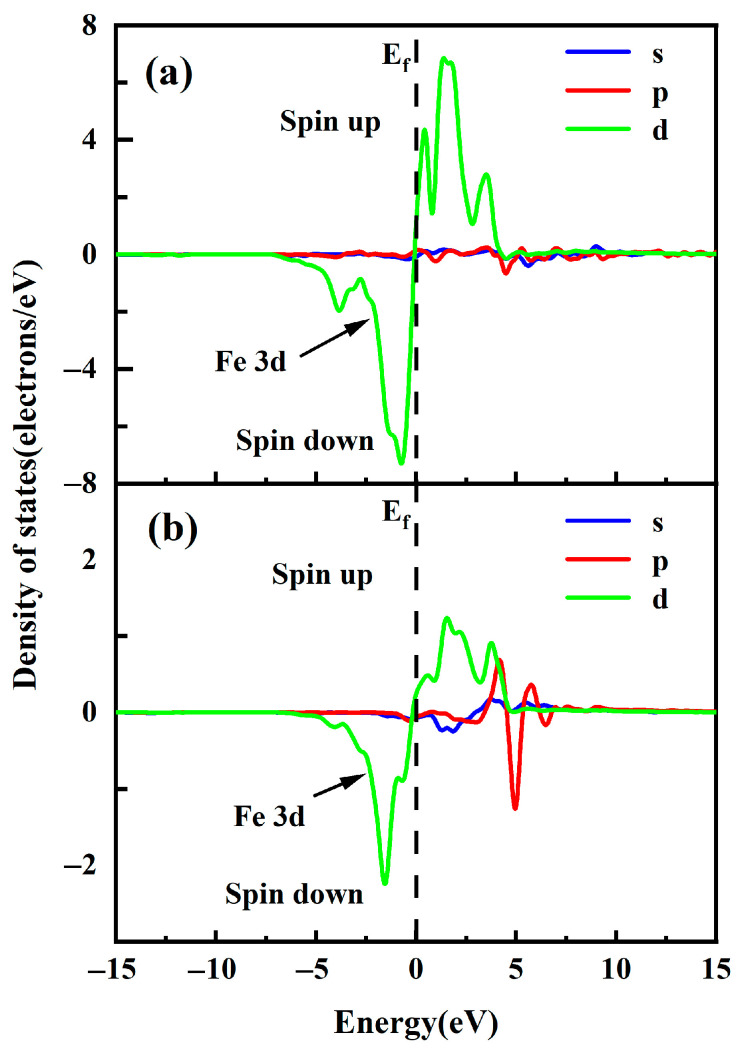
DOS of Fe atom on pyrrhotite (001) surface: (**a**) pyrrhotite top layer and (**b**) free Fe^2+^.

To further investigate the effect of the magnetic properties of free Fe^2+^ on pyrrhotite, we calculate the spin DOS of free Fe^2+^ and the spin DOS of pyrrhotite. As shown in Figure 4, the spin DOS of the Fe atom in the pyrrhotite (001) top layer is mainly contributed by the Fe3d orbital. It shows some symmetry in the range −5eV~5eV, but the down-spin energy of the Fe atom at the −5eV to Fermi level is greater than the up-spin energy at the Fermi level to −5eV. Therefore, the Fe atoms of pyrrhotite contribute more negative magnetic moments. The spin DOS of the free Fe^2+^ is mainly contributed by the Fe3d, 4s orbitals. In the range of −5eV to 5eV, there is a certain asymmetry, but the free Fe^2+^ is the same as the Fe atoms of pyrrhotite (001), with spin-down energy greater than spin-up energy. Therefore, the free Fe^2+^ also contributes more negative magnetic moments.

The spin densities of free Fe^2+^, pyrrhotite (Fe) with free Fe^2+^ added, and pyrrhotite (Fe) without free Fe^2+^ added were further determined, as shown in Table 1. The spin density of both is negative due to the Fe atom providing more negative magnetic moments. When spin density and |spin density| are not 0 and |spin density| is greater than spin density, it is ferrimagnetic. Due to the total spin polarization of pyrrhotite with Fe^2+^ lower than that of pure pyrrhotite, the overall magnetism of pyrrhotite surface is ferrimagnetic, whereas the pyrrhotite-Fe^2+^ interaction is antiferromagnetic. In addition, when the spin density and |spin density| are approximately 0, it is paramagnetic. Therefore, the free Fe^2+^ is paramagnetic and remains in a high spin state. This phenomenon indicates that the addition of Fe^2+^ affects the magnetic properties of pyrrhotite, which in turn leads to two different types of magnetic properties.

### 2.5. Adsorption of Xanthate on the Surface of Pyrrhotite (001)

**Figure 5:**
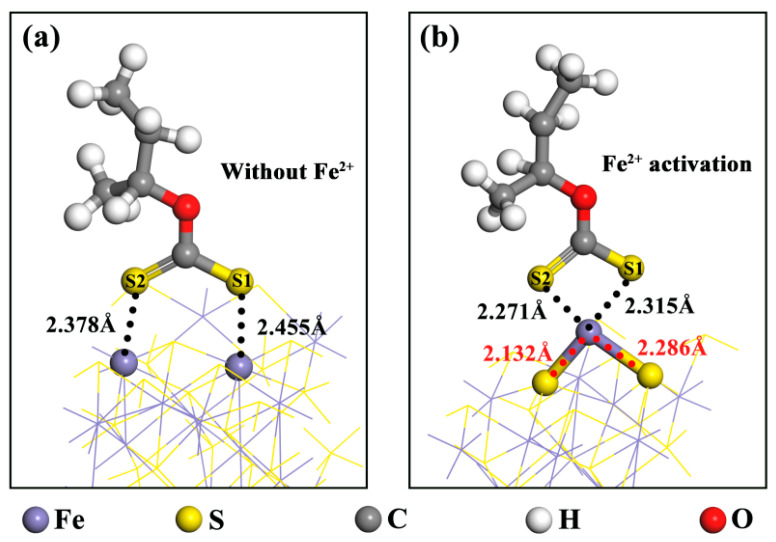
Adsorption models of BX on the pyrrhotite (001) surface: (**a**) without Fe^2+^ activation (**b**) with Fe^2+^ activation.

The adsorption models of BX on the surface of pyrrhotite (001), both without Fe^2+^ activation and following Fe^2+^ activation, are illustrated in Figure 5. The adsorption energies of BX are recorded at −154.6 kJ/mol and −287.7 kJ/mol, respectively. Additionally, their atomic radii are all below 2.6 Å, aligning with the maximum atomic radii of Fe^2+^ and S. These results indicate that BX possesses a strong adsorption capacity for pyrrhotite, and the adsorption effect of BX on pyrrhotite is notably enhanced with the addition of Fe^2+^. Furthermore, when BX interacts with Fe^2+^-activated pyrrhotite, it alters the activation structure of Fe^2+^ from its initial top adsorption configuration to an interaction with another S atom on the pyrrhotite surface, forming a bridged adsorption structure. This leads to the formation of a stable chelate structure with the two S atoms on BX. The BX structure in the presence of Fe^2+^ activation is more stable compared to BX without Fe^2+^ activation. This stability correlates with the robust activation effect of Fe^2+^ observed in the flotation tests.

### 2.6. DOS Analysis of Fe^2+^ and S in BX

The density of states (DOS) is a crucial parameter in solid-state physics that describes the electronic motion states. It finds a wide range of applications in the fields of solid-state physics, surface science, and interfacial adsorption [28,29,30]. DOS can be represented in several forms. The first is the total density of states, which encompasses contributions from all atomic orbitals in the system. The second form is the partial density of states (PDOS), which provides contributions from individual orbitals. The third form involves projecting the DOS onto the atoms to obtain the local density of states (LDOS) [31].

**Figure 6:**
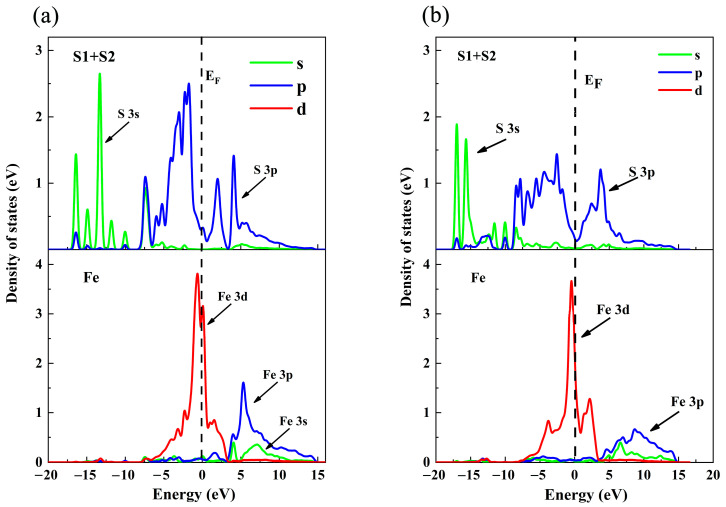
DOS of BX on the surface of pyrrhotite (001): (**a**) Fe^2+^ activation (**b**) without Fe^2+.^

The effect of BX on the surface of pyrrhotite (001) was further analyzed and observed. DOS analysis was performed on two different S atoms (S1 + S2) on BX, the Fe^2+^ on the surface of pyrrhotite and the activated ion Fe^2+^. The results are shown in Figure 6 below.

As observed from Figure 6a, at the Fermi level, both Fe^2+^ and S in BX exhibit orbital contribution energy. Specifically, the primary contribution from Fe^2+^ originates from the 3d orbital, while for S, it is predominantly from the 3p orbital. A comparison of their orbital distributions near the Fermi level reveals a significant overlap between the 3d orbital of Fe^2+^ and the 3p orbital of S in the energy range of −5 to 0 eV, indicating a strong hybridization effect. From Figure 6b, it is evident that the 3d orbit of Fe^2+^ on the pyrrhotite surface also overlaps with the 3p orbit of S in BX within the same energy range of −5 to 0 eV. However, the peak state density for the S of BX in the 3p orbit is at 1.4 eV. In the scenario of Fe^2+^ activation, this peak shifts to 2.5 eV for the S of BX in the 3p orbit. In both instances, BX can adsorb onto the surface of pyrrhotite, leading to the formation of stable chemical bonds. The chemical bond formed between Fe^2+^ and S in BX is stronger than that formed by the direct action of BX on the pyrrhotite surface. This finding aligns with the results obtained from the adsorption analysis.

### 2.7. Mulliken Analysis of Fe^2+^ on the Surface of Pyrite (001)

The Mulliken population, also known as the Mulliken bond population, reflects the overlap of electrons between two atoms and provides a criterion for ionic and covalent bonding between two atoms [32,33]. The Mulliken population Table 2 was obtained by calculating the pyrrhotite model with or without the active ion Fe^2+^.

Table 2 shows that after Fe^2+^ activation, the population values of BX and Fe^2+^ are 0.41 and 0.42, respectively. The similarity in values suggests a considerable charge overlap between the two atoms, indicating a strong covalent nature of the chemical bond and the formation of a stable chemical bond. Additionally, these two chemical bonds collectively create a stable chelating structure. When Fe^2+^ activation is absent, the population values of Fe on the surface of BX and pyrrhotite are found to be 0.25 and 0.27, respectively, which are lower compared to those in the case of Fe^2+^ activation. The S atom in BX interacts with the different Fe atoms on the surface of pyrrhotite, and its structure is less stable than the chelate structure. Evidently, the adsorption of BX on the surface of pyrrhotite is more effective with the addition of Fe^2+^, indicating a significant activation effect of Fe^2+^ on pyrrhotite.

## 3. Materials and Methods

### 3.1. Description of Samples

Pyrrhotite samples were obtained from the Dulong Mining Area, Maguan County, Wenshan Prefecture, Yunnan Province, China. The samples were manually crushed, hand-picked for purification, and subjected to grinding in an agate grinding bowl using a triple-head grinder. Prior to grinding, the grinding bowls were cleaned with quartz sand and anhydrous ethanol. The prepared samples were subjected to dry sieving, and samples with particle sizes from −74 μm to −37 μm were mixed for the subsequent microflotation experiments. The samples were then evacuated and sealed because pyrrhotite is highly susceptible to oxidation.

**Figure 7:**
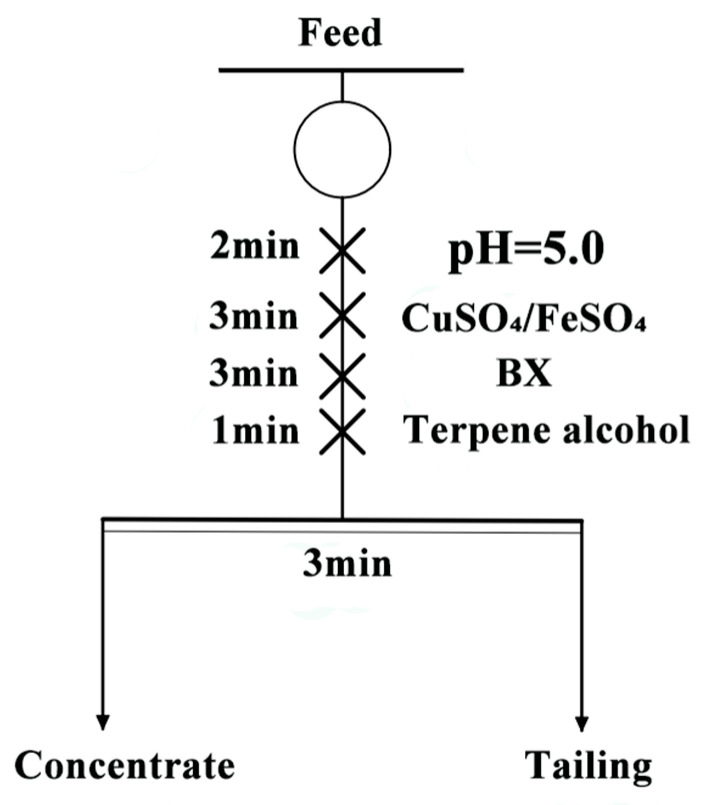
Flowsheet for single-mineral flotation.

Considering that pyrrhotite is a typical sulfide ore, we chose BX, a collector commonly used in sulfide ores to collect pyrrhotite. At the same time, FeSO_4_ and CuSO_4_, two commonly used metal ion activators, were selected to provide the Fe^2+^ and Cu^2+^ explored in the experiment. BX, purchased from Aladdin Industrial Company, Shanghai, China, was used as the collector. Copper sulfate (CuSO_4_) and ferrous sulfate (FeSO_4_), obtained from Tianjin Zhiyuan Chemical Reagent Co., Ltd., Tianjin, China, were used as the activators. Terpene alcohol was used as the foaming agent. Sodium hydroxide (NaOH) and hydrochloric acid (HCl) were used as pH adjusters and activators. All experiments were conducted using deionized water. The experiment process is shown in Figure 7.

### 3.2. Microflotation

In the single mineral flotation experiments, a miniature flotation machine with a capacity of 50 mL was used. The process began with mixing 40 mL of deionized water and 2.0 g of mineral samples in the flotation cell, which were then conditioned for 2 min. The pH of the pulp was adjusted using NaOH and HCl, followed by a further conditioning period of 2 min. The activators, Cu^2+^ and Fe^2+^, were added, along with the collector BX, each being allowed an action time of 3 min. Terpene alcohol was subsequently introduced with an action time of 1 min. Finally, the foam was manually removed using a plastic blade for a duration of 3 min. The concentrate and tailings obtained from the test were filtered, dried, and weighed in a vacuum drying oven at constant temperature.

### 3.3. Calculation Method

**Figure 8:**
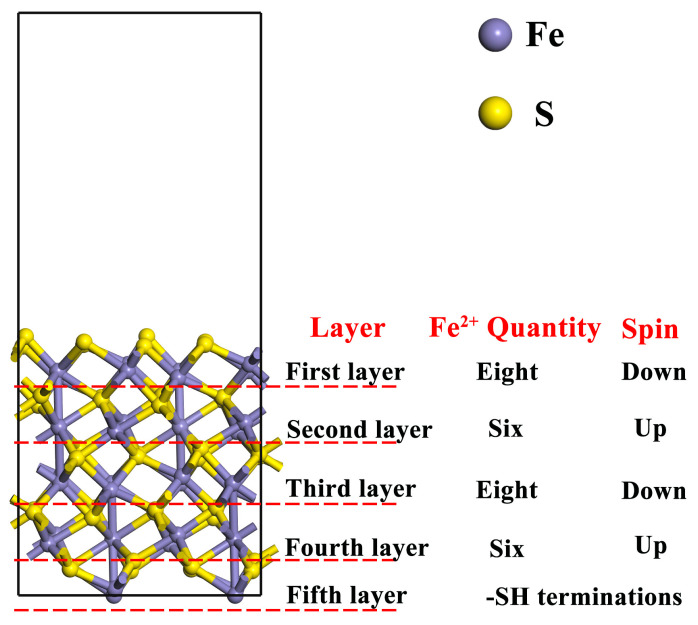
Spin setting of pyrrhotite (001) and the quantity of Fe^2+^ per layer.

The electronic structure and properties of pyrrhotite crystals and surfaces were simulated using DFT in the CASTEP module of Materials Studio 2019 software. Pyrrhotite is a non-stoichiometric compound of the general formula Fe_1−x_S, based on Fe^2+^ and S^2-^ ions. Values for x vary from 0 (FeS) to 0.125 (Fe_7_S_8_). Common pyrrhotite possesses a monoclinic crystal structure with a space group of C2/c [4,34,35,36,37]. Monoclinic pyrrhotite was chosen as the primary model for these calculations based on prior computational experience. The lattice constants of pyrrhotite were initially optimized, employing the PW91 function within the generalized gradient approximation for exchange-correlation generalization. The interaction between valence electrons and the ionic cores on mineral surfaces was described using ultrasoft pseudopotentials. The valence electron configurations for the elements involved in these calculations were set as H 1s1, O 2s2 2p4, S 3s2 3p4, C 2s2 2p2, Fe 3d6 4s2, and Ca 4s2. The plane-wave truncation energy was fixed at 360 eV. Calculations were performed in reciprocal space, using the Monkhorst–Pack scheme for integrals with a k-point grid of 1 × 4 × 1. The self-consistent convergence accuracy was maintained at 2.0 × 10^−5^ eV/atom, with maximum atomic displacement set at 0.002 Å, self-consistency at 2.0 × 10^−5^ eV/atom, the force on each atom limited to 0.08 eV/Å, and internal stress constrained to 0.1 GPa. Considering pyrrhotite’s magnetic properties, the influence of spin was incorporated in all calculations. In the calculation process, the up and down high spin was added to the pyrrhotite model. The amount of Fe^2+^ in each layer was also recorded according to the crystal structure of the pyrrhotite [34,38,39]. Considering the periodic structure of the model, the bottom layer was constrained so that it did not participate in the optimization, and the -SH terminate was added. The optimized model is shown in Figure 8.

**Figure 9:**
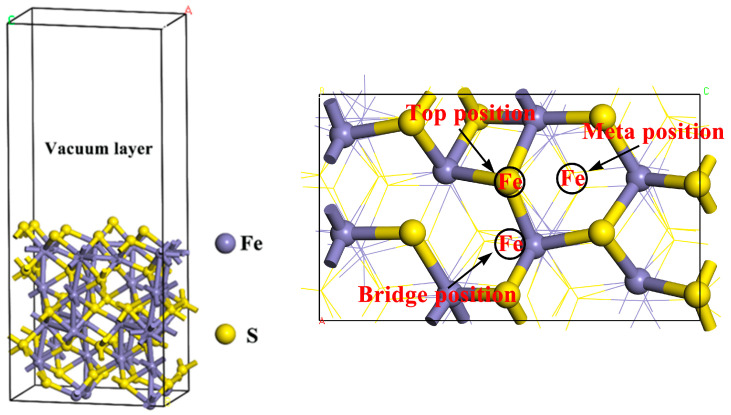
Monoclinic pyrrhotite (001) model after geometric optimization.

Zhao et al.’s study on the interactions of cyanide with pyrite, marcasite, and pyrrhotite found that the (001) surface is the optimal cleavage plane for pyrrhotite [4,37]. A (1 × 2) supercell geometry was modeled for the pyrrhotite (001) surface. Surface energies were computed for various surfaces with differing slab thicknesses to determine the optimal slab size. The most stable surface model, derived from DFT calculations, featured a vacuum layer of 15 Å, as depicted in Figure 9.

**Figure 10:**
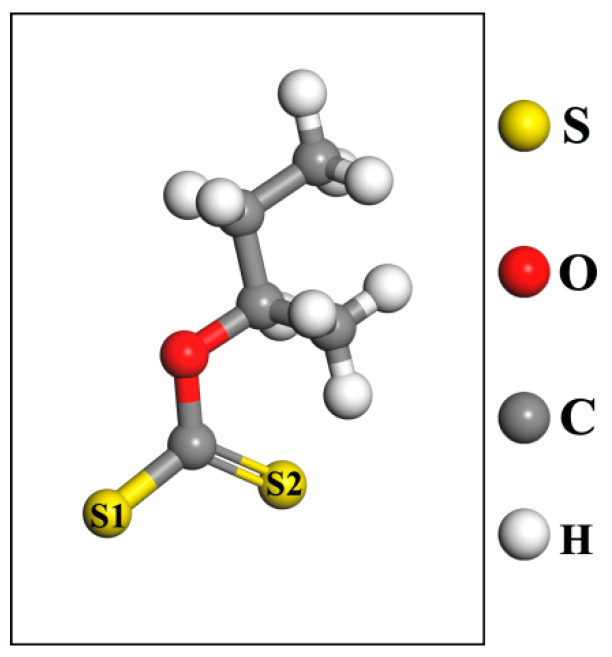
BX model after geometric optimization.

At present, there is no article that specifically studies the optimal adsorption site of Fe^2+^ in pyrrhotite. Therefore, three adsorption sites were selected for comparison. First, the top position on S of monoclinic pyrrhotite was selected as the adsorption site for Fe^2+^; secondly, the bridge position between the two S of monoclinic pyrrhotite was selected as the adsorption site of Fe^2+^. Finally, the meta position between the three S of monoclinic pyrrhotite was selected as the adsorption site of Fe^2+^. Moreover, considering that pyrrhotite is a sulfide mineral, we needed to verify the activation effect of Fe^2+^ on pyrrhotite. BX is a commonly used collector for sulfide minerals, and isobutyl xanthate was selected for subsequent calculations in this calculation, which is also a kind of butyl xanthate, corresponding to the microflotation experiment [40,41,42,43,44]. BX should be added in the future to further study the activation of pyrrhotite by Fe^2+^. The BX model is shown in Figure 10.

The final adsorption energy ($E_{ads}$) is obtained using the following Equation (2):

(2) $$E_{ads}=E_{\left(ads+slab\right)}-(E_{colletor}+E_{slab}+E_{ion})$$

Here, $E_{ads}$ is the final adsorption energy, $E_{\left(ads+slab\right)}$ is the total energy after optimization of the pyrrhotite (001) surface adsorbent (Fe^2+^, BX), and $E_{slab}$ is the energy after optimization of the pyrrhotite (001) surface. $E_{colletor}$ and $E_{ion}$ represent the optimized energies of BX and Fe^2+^, respectively. Adsorption energy indicates the enthalpy change in the reaction between substances. The greater the absolute value of adsorption energy in negative, the easier it is for the adsorption to occur. All quantum mechanical simulation calculations were carried out in a vacuum.

## 4. Conclusions

In the case of a BX dosage of 60mg/L and flotation pH = 5, with an increase in CuSO_4_ dosage from 0 to 8 × 10^−4^ mol/L, the recovery of pyrrhotite initially rose slightly from 71.27% to 87.65% but subsequently experienced a sharp decline to 16.47%. Conversely, as the FeSO_4_ dosage was augmented from 0 to 8 × 10^−4^ mol/L, the recovery of pyrrhotite increased from 71.27% to 82.37%. These observations indicate a higher sensitivity of CuSO_4_ to dosage variations, suggesting that even minor adjustments in CuSO_4_ dosage can considerably influence its effectiveness under certain experimental conditions. In contrast, FeSO_4_ appears to be less sensitive to changes in dosage, leading to more stable performance. Hence, FeSO_4_ demonstrates potential as a suitable alternative activator to CuSO_4_ for industrial purposes.

Fe^2+^ can chemically adsorb onto the surface of pyrrhotite (001) in the form of the top position, forming a stable chemical bond and exhibiting a pronounced activation effect on pyrrhotite. The introduction of BX and its interaction with Fe^2+^-activated pyrrhotite leads to the formation of four Fe-S bonds on Fe^2+^, with close atomic distances, resulting in a stable double-chelate structure. Although hybridization between S 3p orbitals on BX and Fe 3d orbitals on pyrrhotite is present, the hybrid effect is more pronounced with Fe^2+^ activation. Furthermore, the Fe-S bond created upon adding Fe^2+^ activation displays higher Mulliken population values, more significant charge overlap, and a stronger covalent bond. Consequently, Fe^2+^ is identified as an efficient and stable activator for pyrrhotite.

## Figures and Tables

**Table 1:** Spin density table of free Fe^2+^ and pyrrhotite(Fe).

Model	Spin Density/μ_B_	|Spin Density|/μ_B_	States
Pyrrhotite(Fe) alone	−8.39	25.98	Antiferromagnetic
Pyrrhotite(Fe) with Fe^2+^	−6.02	21.52	Ferrimagnetic
Free Fe^2+^	−4.54 × 10^−8^	1.37 × 10^−7^	Paramagnetic, HS

**Table 2:** Mulliken population table of S and Fe.

Adsorption Model	Chemical Bond	Mulliken Population	Bond Length/(Å)
Pyrrhotite + Fe^2+^ + BX	Fe-S1	0.41	2.315
	Fe-S2	0.42	2.271
Pyrrhotite + BX	Fe1-S1	0.25	2.455
	Fe2-S2	0.27	2.378

## Data Availability

The original contributions presented in the study are included in the article, further inquiries can be directed to the corresponding authors.
